# Pharmacological Activation of Autophagy Restores Cellular Homeostasis in Ultraviolet-(B)-Induced Skin Photodamage

**DOI:** 10.3389/fonc.2021.726066

**Published:** 2021-08-02

**Authors:** Sheikh Ahmad Umar, Naikoo Hussain Shahid, Lone Ahmad Nazir, Malik Ahmad Tanveer, Gupta Divya, Sajida Archoo, Sharma Rai Raghu, Sheikh Abdullah Tasduq

**Affiliations:** ^1^Biological Sciences, Academy of Scientific & Innovative Research (AcSIR), Ghaziabad, India; ^2^Pharmacokinetics-Pharmacodynamics (PK-PD) and Toxicology Division, Council of Scientific & Industrial Research (CSIR)-Indian Institute of Integrative Medicine, Jammu Tawi, India

**Keywords:** ultraviolet radiation (UV-B), oxidative stress, endoplasmic reticulum stress, autophagy, DNA damage response, genotoxicity

## Abstract

Ultraviolet (UV) exposure to the skin causes photo-damage and acts as the primary etiological agent in photo-carcinogenesis. UV-B exposure induces cellular damage and is the major factor challenging skin homeostasis. Autophagy allows the fundamental adaptation of cells to metabolic and oxidative stress. Cellular dysfunction has been observed in aged tissues and in toxic insults to cells undergoing stress. Conversely, promising anti-aging strategies aimed at inhibiting the mTOR pathway have been found to significantly improve the aging-related disorders. Recently, autophagy has been found to positively regulate skin homeostasis by enhancing DNA damage recognition. Here, we investigated the geno-protective roles of autophagy in UV-B-exposed primary human dermal fibroblasts (HDFs). We found that UV-B irradiation to HDFs impairs the autophagy response in a time- and intensity-independent manner. However, improving autophagy levels in HDFs with pharmacological activators regulates the UV-B-induced cellular stress by decreasing the induction of DNA photo-adducts, promoting the DNA repair process, alleviating oxidative and ER stress responses, and regulating the expression levels of key cell cycle regulatory proteins. Autophagy also prevents HDFs from UV-B-induced nuclear damage as is evident in TUNEL assay and Acridine Orange/Ethidium Bromide co-staining. Salubrinal (an eIF_2_α phosphatase inhibitor) relieves ER stress response in cells and also significantly alleviates DNA damage and promotes the repair process in UV-B-exposed HDFs. P62-silenced HDFs show enhanced DNA damage response and also disturb the tumor suppressor PTEN/pAKT signaling axis in UV-B-exposed HDFs whereas *Atg7*-silenced HDFs reveal an unexpected consequence by decreasing the UV-B-induced DNA damage. Taken together, these results suggest that interventional autophagy offers significant protection against UV-B radiation-induced photo-damage and holds great promise in devising it as a suitable therapeutic strategy against skin pathological disorders.

## Introduction

Skin being the external covering of the body protects internal organs from outside environmental insults including the adverse effects of ultraviolet (UV) irradiation (1). Solar radiation is essential for survival to different life forms on earth, but excessive exposure leads to skin photoaging and malignancies constituting photo-damage and photo-carcinogenesis (2). Macro-autophagy (hereafter referred to as autophagy) at basal levels protects cells from stress and nutrient deprivation during starvation conditions and thereby maintains tissue homeostasis (3). Cellular autophagy levels can be improved chemically in order to restore tissue homeostasis in response to diverse physiological and pathological stresses, including from solar UV-B irradiation (4–6). Dysfunctional autophagy has been associated to multiple human pathologies, such as metabolic diseases, cardiovascular diseases, aging, neuro-degeneration, infectious diseases, and cancer, and attempts are being made to use autophagy as a selective therapeutic intervention in different disease conditions based on the differential roles it performs in maintaining tissue homeostasis (7, 8). The role of autophagy is context dependent and performs both oncogenic and tumor-suppressive functions (9), promoting or suppressing tumorigenesis and thereby regulates inflammation, cell proliferation, and migration (10, 11). Autophagy removes cellular debris to prevent genomic damage or promotes DNA repair in response to ionizing radiation-induced DNA double-strand breaks in mammalian cells (11, 12). In either way, the role of autophagy is to protect cells from external insults that disturb the integrity of cells. Recently, it has been observed that autophagy regulates nucleotide excision repair (NER) and eliminates DNA base lesions, including cyclobutane pyrimidine dimers (CPD) and pyrimidine-(6-4)-pyrimidone photoproducts (6-4PP) induced by solar UV-B radiation (13–15). It has also been found that recruitment of DDB2 to UV-induced CPD sites is significantly impaired in autophagy-deficient cells. In mice, Rapamycin has been found to decrease the UV-B-induced tumorigenesis while the inhibitor Spautin-1 augments it (16, 17). These findings cite the critical role of autophagy in maintaining proper NER activity and suggest a new tumor-suppressive mechanism of autophagy in tumor initiation and regulation. Previously, we have reported from our own lab that UV-B-induced Ca^2+^ deficit within ER lumen is mediated by immediate oxidative stress induced upon UV-B irradiation to skin cells. Insufficient Ca^2+^ reserves within ER lumen develops ER stress leading to Unfolded Protein Response (UPR) in skin cells that ultimately disturb the cellular homeostasis (18). We have also reported in another study that the natural anti-oxidant bio-active molecule Glycyrrhizic acid (GA) alleviates oxidative stress-induced DNA damage response (DDR) by improving cellular autophagy signaling in UV-B-irradiated primary HDFs (19). Despite these preliminary findings, the role of autophagy in UV-B-induced photo-damage response is unclear and warrants further studies to unravel the facts. In line with these previous findings, we hypothesized the possible integration of DDR and autophagy signaling axis as a positive association in protecting skin cells against genotoxic stress response (20). Here, in this study, we attempted to investigate the roles of autophagy in regulating skin homeostasis notably under genotoxic stress on UV-B radiation exposure to HDFs. We found that UV-B irradiation to HDFs induce impaired autophagic flux at a lethal dose of UV-B irradiation (30 mJ/cm^2^). However, improving autophagy response with pharmacological activator Rapamycin significantly alleviates the induction of oxidatively induced DNA photo-adducts and enhances the DNA repair mechanism, alleviates the TUNEL-positive cells, and reduces early and late apoptotic cells and prevents ER calcium leakage in HDFs in 6 h UV-B post-irradiation. Furthermore, we found that relieving ER stress response with Salubrinal prevents oxidative DNA damage by improving autophagy response in UV-B-exposed HDFs. The GFP-RFP-LC3B puncta assay depicts appreciable red puncta dots in Rapamycin- and Salubrinal-treated cells showing enhanced autophagic flux upon UV-B exposure to HDFs compared to those exposed only to UV-B. Rapamycin treatment also significantly decreases the expression profile of key cell cycle regulatory proteins P21 and P27 and DDR pathway proteins DDB2 and p-P53, indicating that autophagy has cell protective roles in UV-B-induced photo-damage. Decreasing autophagic flux by silencing P62 confirmed our preliminary findings as the p-χH_2_AX foci are significantly augmented in P62-silenced cells but not in *Atg7*-silenced HDFs citing differential roles of autophagy-related genes in regulating UV-B-induced genotoxic stress response. Together, the above findings suggest that pharmacological activation of autophagy significantly alleviates the DNA damage and promotes the DNA repair process in UV-B-exposed HDFs and is critical in restoring cellular homeostasis. Furthermore, these results suggest that interventional autophagy holds great promise to be devised as a suitable therapeutic strategy against radiation-induced skin photo-damage disorders.

## Materials and Methods

### Chemicals

Human primary dermal fibroblast cell line from juvenile foreskin (HDF) and primary fibroblast expansion media was obtained from HiMedia, Mumbai, India. Fetal bovine serum (FBS), penicillin–streptomycin, trypsin–EDTA, 3-(4, 5-dimetylthiazol-yl)-diphenyl tetrazolium bromide (MTT), phosphatase-protease cocktail, RIPA buffer, and H_2_DCFDA dye were purchased from Sigma–Aldrich Chemicals (St. Louis, MO). Antibodies against P62, BECN1, phospho-ATM, phospho-ATR, phospho-mTOR, phospho-p53, phospho-Chk1, phospho-Chk2, Bcl-2, phospho-eIF_2_α, eIF_2_α, LC3B, phospho-AMPKα, AMPK, and phospho-χH_2_AX were purchased from Cell Signaling Technology, Danvers, MA. Antibodies against GRP78, PTEN, CHOP/GAD153, DDB2, phospho-AKT, and siRNA P62/*Atg7* and secondary antibodies were purchased from Santa Cruz Biotechnologies (Santa Cruz, CA, USA). Fura 3 AM, DAPI, ER tracker, Acridine Orange, Ethidium Bromide, Salubrinal, Rapamycin, Chloroquine, Bafilomycin A1, Everolimus, and GFP-RFP-LC3B puncta assay kit were purchased from Thermo Scientific. Antibodies against P21 and P27, TUNEL assay, and CPD ELISA kits were procured from Abcam. Bradford reagent was obtained from Sigma-Aldrich. PVDF membrane was purchased from Bio-Rad, Hercules, CA. Antibody against β-actin was purchased from Sigma-Aldrich.

### Cell Culture and UV-B Exposure to HDFs

HDFs were maintained in primary fibroblast expansion media from HiMedia supplemented with all the essentials including antibiotics, L-Glutamine, glucose (3.5 g/L), Hepes (15 mM), Penicillin (120 mg/L), Streptomycin (270 mg/L), and FBS (10% v/v) at 37°C in a humidified atmosphere of 5% CO_2_. Cells were exposed to UV-B using DAAVLIN UVA/UVB Research Irradiation Unit (Bryan, OH, USA) having digital control. The lamps were maintained at a fixed distance of 24 cm from the surface of cell culture dishes. Majority of the resulting wavelengths (>90%) were in the UV-B range (280–320 nm). UV-B irradiation of 10, 20, and 30 mJ/cm^2^ was used for initial standardization and dose optimization and 30 mJ/cm^2^ dose was then selected and used for further experiments for mechanistic studies based on the analysis of cell toxicity induced by UV-B exposure to HDFs. Though 10 mJ/cm^2^ is considered as the physiological dose mimicking the environmental dosage of UV-B in the solar radiation spectrum (21), it induces less cytotoxicity (10%–20%) and shows least molecular changes to be selected for mechanistic studies. Before UV-B exposure, cells were first sensitized with chemical mediators like Rapamycin, Chloroquine, Salubrinal, Bafilomycin, and siRNA P62/*Atg7* for a specified time period as per the particular experimental requirements to induce or inhibit autophagy. Cell monolayers were then first washed with Dulbecco’s phosphate buffered saline (DPBS) and then UV-B-irradiated under a thin layer of pre-warmed DPBS. After irradiation, cells were again washed with DPBS twice and incubated in fresh medium with or without chemical mediators as per the experimental protocol requirements for 1, 3, 6, or 24 h UV-B post-irradiation.

### Cell Viability Analysis

Colorimetric-based MTT assay was employed for cell viability analysis as described earlier (22). Briefly, cells were seeded and incubated overnight in a humidified chamber. After treatment with autophagy modulators or UV-B or both, the cells were further incubated for 24 h. Cell viability was evaluated by assaying for the ability of functional mitochondria to catalyze the reduction of MTT to formazan salt by an enzyme mitochondrial dehydrogenase that appears as crystals at the bottom of culture wells/dishes and was quantified by a MULTISKAN SPECTRUM plate reader (Thermo Electron Corporation) at 570 nm using DMSO as solvent. The mean of three independent readings was taken for final quantification of data for result analysis.

### Determination of Reactive Oxygen Species

Dichlorofluorescin Diacetate (H_2_DCF-DA) staining was employed for the measurement of immediate ROS generated upon UV-B exposure to HDFs (1 h) post-UV-B irradiation, as described previously (21). Briefly, HDFs were seeded in six-well plates and allowed to attach overnight. Cell monolayers were pre-treated with Salubrinal (25 µM) and Everolimus (200 nM) for 2 h. Cells were washed three times with DPBS and then exposed to UV-B 30 mJ/cm^2^. After UV-B irradiation, cells were then again washed with DPBS twice and incubated with fresh media with Salubrinal (25 µM) and Everolimus (200 nM) for 1 h UV-B post-irradiation. After treatment, the cells were stained with 5 μM H_2_DCF-DA for 30 min at 37°C. The cells were then washed with DPBS thrice and observed immediately under a fluorescent microscope (EVOS FL Color Imaging System from Life Technologies, B2014-155G-054). Five random microscopic fields were selected and the intensity of fluorescence was quantified using the ImageJ software, as mentioned previously (23).

### Confocal Microscopy Imaging Of Intracellular Ca^2+^ Release

Ca^2+^ levels were determined by the Ca^2+^ indicator Fura 3 AM (Thermo Scientific) using confocal microscopy imaging as described previously. Briefly, the cells were seeded to sterile coverslips and incubated overnight in a humidified chamber to adhere. Cells were or were not treated with Salubrinal (25 µM), Rapamycin (100 nM), and Bafilomycin A1 (100 nM) for 2 h. Cells were then thrice washed with DPBS and exposed to UV-B treatment at 30 mJ/cm^2^ as described earlier and supplemented with fresh DMEM media with indicated concentrations of Salubrinal, Rapamycin, and Bafilomycin as required and incubated further for 6 h post-UV-B irradiation. HDFs were loaded with fluorescent Ca^2+^ indicator dye Fura 3 AM at 5 µM for 45 min before imaging post 6 h UV-B irradiation. Cells were washed three times with live cell imaging solution for imaging using a laser scanning confocal microscope (OLYMPUS FUOVIEW FV1000) by using a 40× objective lens. Five random microscopic fields were selected, and the intensity of fluorescence was quantified using the ImageJ software.

### siRNA-Mediated Knockdown of P62/*Atg7*

Validated siRNA P62/*Atg7* were purchased from Santa Cruz Biotechnology. siRNA and Lipofectamine (Invitrogen) were diluted in Opti-MEM I reduced serum medium (Invitrogen) per the manufacturer’s instructions. HDFs were incubated for 16 h with transfection mixture at a final siRNA concentration of 50 pmol as described previously (24) and then exposed to UV-B (30 mJ/cm^2^) and were finally supplemented with fresh medium for further 6 h UV-B post-irradiation.

### Protein Isolation and Western Blotting

Cells were trypsinized, harvested in PBS (pH 7.4), centrifuged, and resuspended in RIPA buffer (Sigma-Aldrich). After incubation for 45 min at 4°C, cell lysates were centrifuged at 17,530*g* for 30 min at 4°C to remove cellular debris. Protein concentrations were determined by Bradford reagent. For Western blotting, 30–80 μg protein loads were denatured at 100°C for 3 min in Laemmli buffer. Protein samples were resolved on 4%–15% SDS gels at 70–80 V. Proteins were electro-transferred to PVDF membrane using a *BIO-RAD* Mini Transblot Electrophoretic Transfer unit. Membranes were blocked in 5% fat-free dry milk/3% BSA in 50 mM Tris, pH 8.0, with 150 mM sodium chloride, 2.6 mM KCl, and 0.05% Tween20 for 2 h. Primary antibodies were used either in fat-free milk or BSA and incubated overnight at 4°C. Anti-GRP78, anti-SQSTM1/p62, anti-Bcl_2_, anti-p-mTOR, anti-mTOR, anti-CHOP/GAD153, anti-p-elf_2_α, anti-elf_2_α, anti-BECN1, anti-p-ATM, anti-p-ATR, anti-p-P53, anti-p-Chk1, anti-LC3B, anti-p-AMPKα, anti-AMPKα, anti-p-χH_2_AX, anti-PTEN, anti-p-AKT, and anti-*Atg7* were from Cell Signaling Technology, Danvers, MA, and mouse and anti-actin were from Sigma-Aldrich. Goat anti-rabbit and goat anti-mouse immunoglobulin G antibodies conjugated with HRP (Santa Cruz Biotechnologies) were used as secondary antibodies. Chemiluminescence was detected by Immobilon chemiluminescent HRP substrate (EMD-Millipore, Billerica, MA) and visualized by Molecular Image ChemiDocTM XRS+ (BIO-RAD), Universal Hood II, Serial No 721BR04356. Densitometric measurement of the bands was performed using Image LabTM software (version 3.0; Bio-Rad).

### TUNEL Assay

TUNEL assay was performed with an In Situ Direct DNA Fragmentation (TUNEL) Assay Kit (ab66108) from (Abcam) according to the manufacturer’s instructions as described previously (19). Briefly, the cells were seeded in dishes and incubated overnight in a humidified chamber to adhere. Cells were or were not treated with Salubrinal (25 µM), Rapamycin (100 nM), and Chloroquine (50 µM) for 2 h. Cell monolayers were then washed thrice with DPBS and exposed to UV-B (20 and 30 mJ/cm^2^). Again, the cells were washed thrice with DPBS and supplemented with fresh DMEM media with or without the indicated concentrations of Salubrinal, Rapamycin, and Chloroquine as required and incubated further for 6 h post-UV-B irradiation. Cell smears after fixation, blocking, and permeabilization were incubated with TUNEL reaction mixture for 1 h and wrapped in aluminum foil to avoid light exposure at 37°C and counterstained with RNase/PI solution for an additional 20 min. Substrate solution was added, and cells were imaged by a fluorescent microscope (EVOS FL Colour Imaging System) for the detection of TUNEL-positive cells, and the intensity of fluorescence was quantified using the ImageJ software.

### ELISA-Based Detection of DNA Photo-Adducts

UV-B-induced CPD/6,4 PP photo-adducts were quantified with an *in situ* OxiSelect UV-Induced DNA Damage staining kit (CPD/6,4PP quantification kit from Cell Bio Labs, Inc., San Diego, CA, USA) according to the manufacturer’s instruction. Briefly, the cells were seeded and allowed to adhere overnight. Cells were or were not treated with Salubrinal (25 µM), Rapamycin (100 nM), and Chloroquine (50 µM) for 2 h or were silenced for P62 using siRNA. After the corresponding treatments to HDFs, cell monolayers were exposed to UV-B at (10, 20 and 30 mJ/cm^2^) and supplemented with fresh DMEM media with or without the indicated concentrations of Salubrinal, Rapamycin, and Chloroquine for further 6 h post-UV-B irradiation. DNA was isolated and incubated with the anti-CPD antibody overnight on an orbital shaker at room temperature. Then, the cells were washed and incubated with Secondary Antibody-HRP Conjugate for 2 h. The absorbance was measured at 450 nM using the MULTISKAN SPECTRUM plate reader (Thermo Electron Corporation). A mean of three independent readings was used to quantify the data for final result analysis.

### Dual Acridine Orange/Ethidium Bromide Fluorescent Staining for Detection of Apoptotic Cells

Briefly, the cells were seeded in six-well plates and allowed to adhere overnight in an incubator. Cells were left untreated or treated with Salubrinal (25 µM), Rapamycin (100 nM), and Chloroquine (50 µM) for 2 h and washed thrice with DPBS. Cells were then exposed to UV-B at 30 mJ/cm^2^ and again washed thrice with DPBS. Cells were then supplemented with fresh DMEM media with or without indicated concentrations of Salubrinal, Rapamycin, and Chloroquine as explained for an additional 6 h post-UV-B irradiation. Dual fluorescent staining solution (1 μl) containing 100 μg/ml AO and 100 μg/ml EtBr was added to cell monolayers for 5 min at RT and then covered with coverslip and were fixed as described previously (25). The morphology of HDFs into early and late apoptotic cells was examined within 20 min using a fluorescent microscope (EVOS FL Colour Imaging System). AO/EtBr staining method was repeated at least three times for quantification and the data are presented by the classification of cells to live cells, early apoptotic, late apoptotic, and necrotic cells as reported previously (26).

### Immunostaining

Cultured cells were seeded on coverslips in six-well plates and incubated in the presence or absence of indicated concentrations of Salubrinal (25 µM), Rapamycin (100 nM), and Chloroquine (50 µM) for 2 h. Cells were then exposed to UV-B (30 mJ/cm^2^) and again washed thrice with DPBS and supplemented with fresh DMEM media with indicated concentrations of Salubrinal, Rapamycin, and Chloroquine for required post UV-B time intervals of 1, 6, and 24 h and were fixed in 4% paraformaldehyde for 15 min at room temperature. Cells were permeabilized in PBS containing 0.1%Triton X-100 at room temperature for 10 min. Non-specific binding sites were blocked by incubating the cells with 1% BSA and 22.52 mg/ml Glycine in PBST (PBS+0.1% Tween 20) at room temperature for 30 min. Cells were incubated with P62, LC3B, p-P53, p-χH_2_AX, PTEN, p-AKT, DDB2*, Atg7*, p-AMPKα, P21, and P27 antibodies at a dilution of 1:100 in 1% BSA and 22.52 mg/ml Glycine in PBST (PBS+0.1% Tween 20) overnight at 4°C. Cells were later on washed and incubated with Alexa Fluor 488/594-conjugated antimouse/antirabbit secondary antibody as required at a dilution of 1:500 in 1% BSA and 22.52 mg/ml Glycine in PBST (PBS+0.1% Tween 20) for 2 h at room temperature in dark conditions. Cells were then washed three times with PBS and stained with DAPI 1 μg/ml in PBS. The coverslips were mounted on glass slides, and cells were imaged by a laser scanning confocal microscope (OLYMPUS FUOVIEW FV1000) by using a ×40 objective lens. Five random microscopic fields were selected, and the intensity of fluorescence was quantified using the ImageJ software.

### GFP-RFP-LC3B Puncta Assay for the Detection of Autophagic Flux

For analysis of autophagosomes, the cells were seeded in dishes and allowed to adhere overnight in an incubator. BacMam 2.0 RFP-GFP-LC3B reagent was added to HDFs and incubated overnight to ensure maximum protein expression. The cells were thrice washed with DPBS. Then, the cells were or were not treated with Rapamycin (100 nM), Salubrinal (25 µM), and Bafilomycin (100 nM) for 2 h. The cells were then exposed to UV-B (30 mJ/cm^2^) and again washed thrice with DPBS and supplemented with fresh DMEM media with indicated concentrations of Rapamycin, Salubrinal, and Bafilomycin for an additional 6 h post UV-B irradiation. Cells were then again washed with DPBS thrice and visualized using standard GFP (green fluorescent protein) and RFP (red fluorescent protein) settings. The punctae dots were imaged by a laser scanning confocal microscope (OLYMPUS FUOVIEW FV1000) by using a ×40 objective lens. Five random microscopic fields were selected, and quantification of punctae dots was done as reported previously (27).

### Statistical Analysis

Data are expressed as the mean ± standard deviation (SD). INSTAT statistical software was used to perform statistical analysis. Data are presented as mean ± SE from three independent experiments. Comparison between two groups was performed by Student’s *t*-test and that among groups was carried out by one-way ANOVA for statistical significance. *p* ≤ 0.05, *p* < 0.01, and *p* < 0.001 were considered as statistically significant.

## Results

### UV-B Exposure to HDFs Induce Impaired Autophagy Response

**Figure 1:**
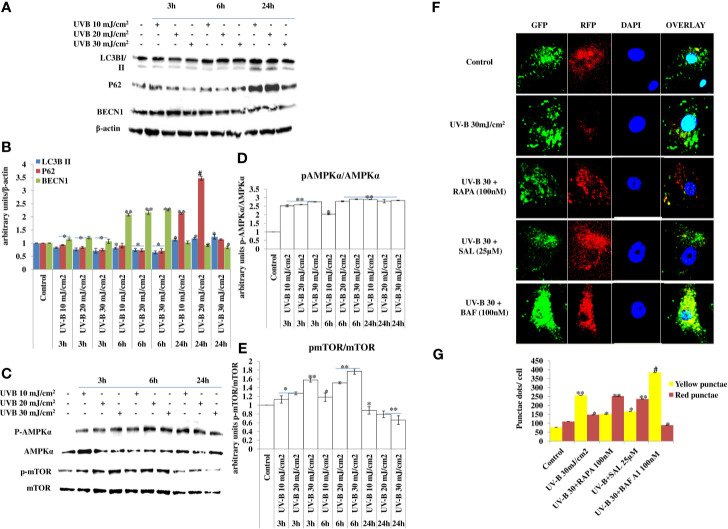
UV-B exposure to HDFs induce impaired autophagy response. **(A–E)** Western blotting analysis of key autophagy marker proteins showing impaired autophagy response in a time- and intensity-independent manner in UV-B 10, 20, and 30 mJ/cm^2^ exposed HDFs. **(F, G)** GFP-RFP-LC3B puncta assay depicting yellow and red punctae dots representative of autophagosomes and autolysosomes in UV-B 30 mJ/cm^2^ exposed HDFs and effect of Rapamycin (100 nM), Salubrinal (25 µM), and Bafilomycin A1 (100 nM) on autophagic flux in 6 h UV-B post-irradiation to HDFs (*p ≤ 0.05, ^**^p ≤ 0.01, ^#^*p* ≤ 0.001 were considered as statistically significant). Western blots were analyzed using Image Lab.exe 3.0.0.39529 and micrographs with ImageJ.exe 1.8.0_172 software.

UV-B irradiation induces impaired autophagy response in a dose-independent manner as is evident from the Western blotting analysis of key autophagy marker proteins. UV-B exposure to HDFs increases the expression levels of LC3BII at 24 h but not at 3 and 6 h, downregulates P62 expression initially in an insignificant manner but not at 24 h, and increases the expression of BECN1 in an altered fashion, which increases immediately after UV-B exposure, but the expression is blocked in delayed post-exposure, which does not correspond to induction of autophagy response. Similarly, the expression levels of p-AMPKα increases in 3 and 6 h UV-B exposure but starts decreasing in 24 h post-UV-B irradiation. The expression of p-mTOR also increases dose dependently at 3 and 6 h UV-B post-irradiation but not at 24 h, indicating induction of impaired autophagy response upon UV-B exposure to HDFs, because the expression status of different autophagy proteins is modulated in an altered fashion not corresponding to normal autophagy response (**Figures 1A–E**). We confirmed the induction of impaired autophagic flux response upon UV-B 30 mJ/cm^2^ exposure to HDFs through GFP-RFP-LC3B puncta assay in confocal microscopy depicting appreciable red puncta dots, indicative of autolysosomes compared to yellow puncta dots indicative of autophagosomes in Rapamycin- and Salubrinal-treated cells showing enhanced autophagy flux upon UV-B exposure to HDFs compared to those exposed only to UV-B. Bafilomycin A1 treatment to UV-B-exposed HDFs was used as a positive control (**Figures 1F, G**).

### Improving Autophagy and Relieving ER Stress Response Alleviates DNA Photo-Adducts (CPD and 6,4PP) in UV-B-Exposed HDFs

**Figure 2:**
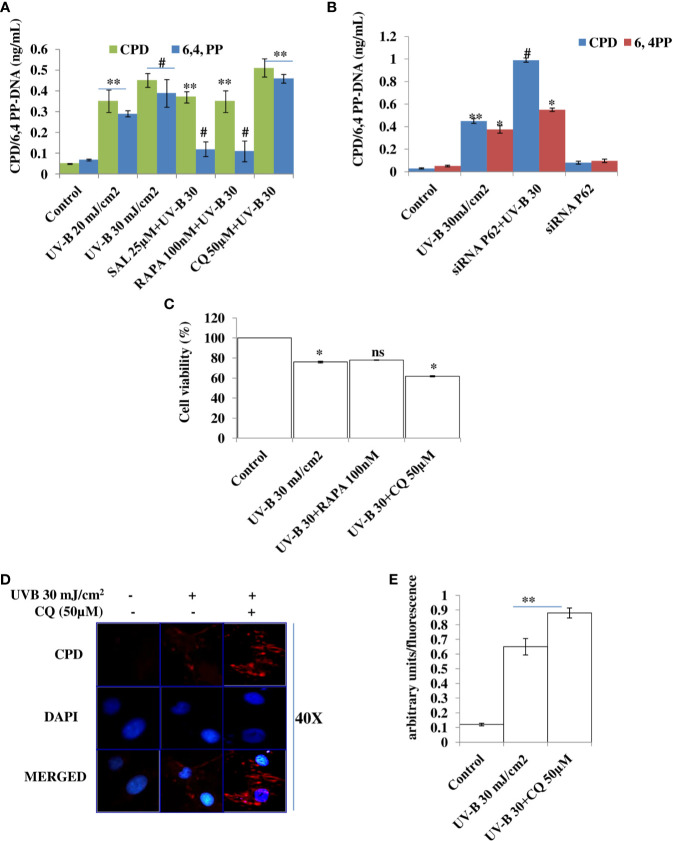
Improving autophagy and relieving ER stress response alleviates DNA photo-adducts in UV-B-exposed HDFs. **(A)** ELISA based quantification of CPD and 6,4PP in UV-B 20–30 mJ/cm^2^ exposed HDFs in 6 h UV-B post-irradiation and effect of Salubrinal (25 µM), Rapamycin (100 nM), and Chloroquine (50 µM) on UV-B-induced CPD and 6,4PP levels. **(B)** ELISA-based quantification of CPD and 6,4PP in siRNA P62-treated HDFs. **(C)** Cell viability analysis of UV-B 30 mJ/cm^2^ exposed HDFs treated with Rapamycin (100 nM) and Chloroquine (50 µM). **(D, E)** Immunofluorescence analysis of CPD protein levels in microscopy in UV-B 30 mJ/cm^2^ exposed HDFs treated with Chloroquine (50 µM) (^*^p ≤ 0.05, ^**^ *p* ≤ 0.01, ^#^ *p* ≤ 0.001 were considered as statistically significant). Western blots were analyzed using Image Lab.exe 3.0.0.39529 and micrographs with ImageJ.exe 1.8.0_172 software. ns. non-significant.

UV-B irradiation to HDFs leads to induction of DNA photo-adducts (CPD and 6, 4PP) in an intensity-dependent manner in 6 h UV-B post-irradiation. Improving autophagy in HDFs with Rapamycin (100 nM) and upon relieving ER stress response with Salubrinal (25 µM) in UV-B-irradiated HDFs significantly alleviates the induction of both CPD and 6,4PP photo-adducts by 0.65- and 0.5-fold, respectively. Inhibition of autophagy response with Chloroquine (50 µM) increases the formation of CPD by 0.2-fold, whereas it increases the formation of 6,4PP by about 0.35-fold compared to UV-B-treated HDFs (**Figure 2A**). To confirm our findings whether pharmacological activation of autophagy improves the UV-B-induced photo-damage response in HDFs, we silenced key autophagy cargo protein P62 and found that it significantly impacts the induction of photo-adducts, CPD, compared to that of 6,4PP and augments the induction of CPD by 0.5-fold and 6,4PP by 0.22-fold. siRNA P62 only treated cells had negligible effect on the induction of DNA photo-adducts compared to both control and UV-B-treated cells (**Figure 2B**). We confirmed ELISA-based findings through immunofluorescence and found that the expression levels of CPD are significantly increased in UV-B-exposed HDFs in an intensity-dependent manner in 6 h UV-B post-irradiation. Inhibiting autophagy response with Chloroquine increases the expression of CPD in immunofluorescence (**Figures 2D, E**). To check whether increasing the autophagy level in HDFs has any effect on the cellular viability in UV-B-irradiated HDFs, we found that UV-B 30 mJ/cm^2^ decreases the cell viability by 25% compared to control. Rapamycin treatment has no significant effect on restoring the cellular viability in UV-B-irradiated HDFs at UV-B 30 mJ/cm^2^ acute dose whereas Chloroquine treatment significantly reduces the cell viability by 0.8-fold compared to those exposed only to UV-B (**Figure 2C**).

### Pharmacological Activation of Autophagy Alleviates Apoptosis and Nuclear Damage in UV-B-Exposed HDFs

**Figure 3:**
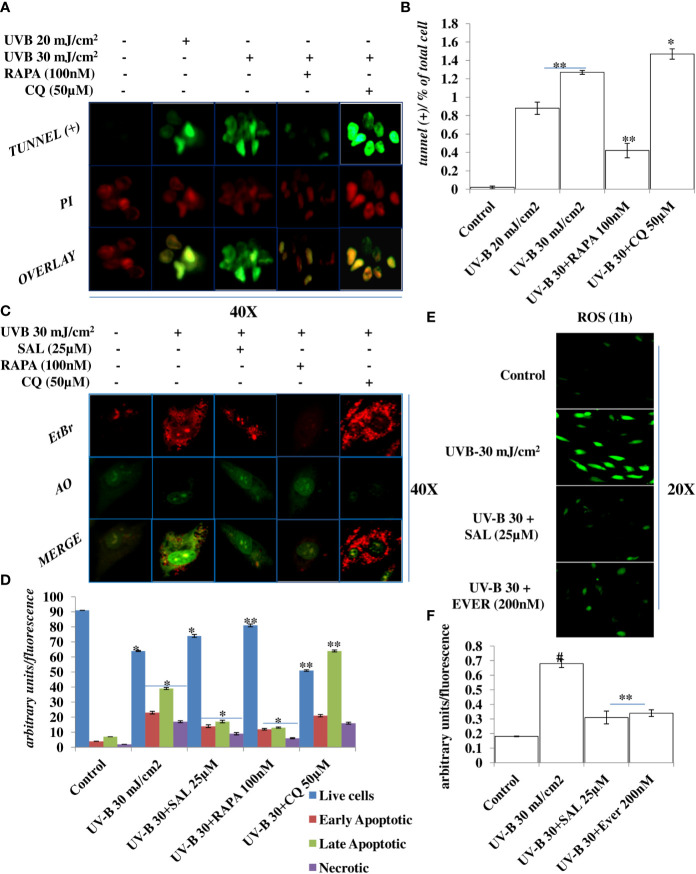
Improving autophagy alleviates apoptotic cells in UV-B-exposed HDFs in fluorescent microscopy. **(A, B)** Fluorescent microscopic analysis of TUNEL (+) cells in UV-B 30 mJ/cm^2^ exposed HDFs showing nuclear damage in 6 h UV-B post-irradiation and effect of Rapamycin (100 nM) and Chloroquine (50 µM) on UV-B-induced TUNEL (+) cells. **(C, D)** Acridine orange and Ethidium Bromide (AO-EtBr) co-staining depicting early and late apoptotic (+) cells in UV-B 30 mJ/cm^2^ exposed HDFs in 6 h UV-B post-irradiation and effect of Salubrinal (25 µM), Rapamycin (100 nM), and Chloroquine (50 µM) on UV-B-induced apoptosis. **(E, F)** Reactive Oxygen Species (ROS) estimation in UV-B 30 mJ/cm^2^ exposed HDFs treated with Salubrinal (25 µM) and Everolimus (200 nM) in 1 h UV-B post-irradiation (^*^p ≤ 0.05, ^**^ *p* ≤ 0.01, ^#^ *p* ≤ 0.001 were considered as statistically significant). Micrographs were analyzed using ImageJ.exe 1.8.0_172 software.

Oxidatively induced DDR is the hallmark of UV-B-induced skin pathologies. To check whether improving autophagy levels in UV-B-irradiated HDFs could alleviate the nuclear alterations by alleviating apoptotic like events in cells, we performed TUNEL assay and Acridine Orange/Ethidium Bromide co-staining. We found that UV-B treatment of HDFs induces TUNEL-positive cells in an intensity-dependent manner in 6 h UV-B post-irradiation. Rapamycin treatment (100 nM) significantly alleviates the fluorescence of TUNEL-positive cells in UV-B 30 mJ exposed HDFs by threefold, whereas Chloroquine treatment (50 µM) of HDFs increases the fluorescence of TUNEL-positive cells by 0.2-fold compared to those exposed only to UV-B 30 mJ (**Figures 3A, B**). In AO/EtBr co-staining, we found that UV-B 30 mJ/cm^2^ irradiation increases early as well as late apoptotic cells in microscopic studies, indicating induction of nuclear damage. Salubrinal 25 µM treatment significantly increases live cells and decreases apoptotic cells by 0.4-fold compared to those irradiated only to UV-B. Similarly, Rapamycin treatment also significantly decreases apoptotic cells in fluorescent microscopy by half compared to those exposed only to UV-B. On the other hand, Chloroquine treatment of HDFs significantly increases early apoptotic and late apoptotic cells by twofold but not necrotic cells compared to those irradiated only to UV-B (**Figures 3C, D**). ROS is the main oxidative damage-causing agent in UV-B-exposed HDFs. To check the effect of autophagy inducer Everolimus (200 nM) and Salubrinal (25 µM) on generation of primary ROS species in 1 h UV-B post-irradiation to HDFs, we found that oxidative stress is the immediate event following UV-B 30 mJ/cm^2^ exposure to HDFs (1 h) by increasing ROS species. Salubrinal and Everolimus treatment to UV-B-exposed HDFs significantly alleviate the production of ROS species by half compared to those exposed only to UV-B and offers significant photo-protection to HDFs by regulating oxidative stress-mediated ER stress response (**Figures 3E, F**).

### Improving Cellular Autophagy Response in UV-B-Exposed HDFs Alleviates DNA Damage Whereas Blockage of Autophagy Augments It

**Figure 4:**
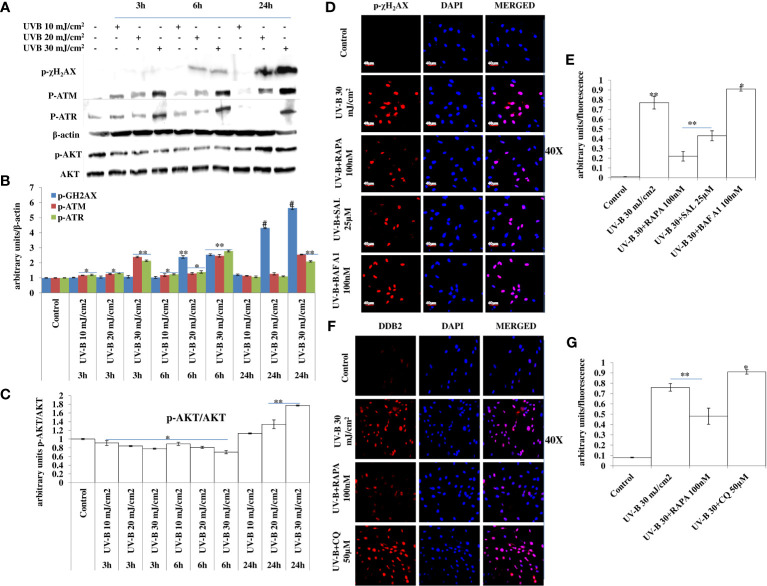
Improving autophagy response alleviates DNA damage in UV-B-exposed HDFs. **(A, B)** Western blotting analysis of DNA damage response proteins showing time- and intensity-dependent effect of UV-B irradiation on DNA damage response proteins in UV-B 10, 20, and 30 mJ/cm^2^ exposed HDFs. **(A, C)** Western blotting analysis of p-AKT protein levels showing the effect of UV-B irradiation on the expression levels of p-AKT decreasing in 3 and 6 h but again augments in 24 h UV-B 10, 20, and 30 mJ/cm^2^ exposed HDFs. **(D, E)** Immunofluorescence analysis of damage sensor protein p-χH_2_AX foci in UV-B 30 mJ/cm^2^ exposed HDFs in 6 h UV-B post-irradiation and effect of Salubrinal (25 µM), Rapamycin (100 nM), and Bafilomycin A1 (100 nM) on p-χH_2_AX expression levels under UV-B exposure to HDFs. **(F, G)** Immunofluorescence analysis of DDB2 protein expression levels in UV-B 30 mJ/cm^2^ exposed HDFs and effect of Rapamycin (100 nM) and Chloroquine (50 µM) on the expression levels of DDB2 (^*^p ≤ 0.05, ^**^ *p* ≤ 0.01, ^#^ *p* ≤ 0.001 were considered as statistically significant). Western blots were analyzed using Image Lab.exe 3.0.0.39529 and micrographs with ImageJ.exe 1.8.0_172 software.

DDR is the natural defense response system activated in cells against any genotoxic stimulus and is attributed at repairing the damaged state to prevent tumorigenesis. Here, we found that UV-B exposure at 10, 20, and 30 mJ/cm^2^ induces DDR in HDFs in an intensity- and time-dependent manner, which is more aberrant in 30 mJ/cm^2^ exposed HDFs as is evident from the Western blotting analysis of key DDR proteins (**Figures 4A, B**). The expression profile of key damage responsive proteins in DNA damage pathway p-χH_2_AX, p-ATM, and p-ATR is significantly modulated in UV-B-exposed HDFs, indicating induction of damage response upon UV-B exposure to HDFs. Moreover, the expression level of p-AKT, which has a crucial role in pro-survival signaling and also inhibits apoptosis in UV-B response is also significantly increased, and the increase is more profound in UV-B 30 mJ/cm^2^ exposed HDFs (**Figures 4A, C**). We confirmed our Western blotting results through immunofluorescence in 6 h UV-B post-irradiation to HDFs in confocal microscopy by imaging for the UV-B-induced p-χH_2_AX foci, which directly reflect the intensity of damage response. In microscopic analysis, we found that UV-B 30 mJ exposure to HDFs induces the expression of p-χH_2_AX foci. Treatment of Rapamycin (100 nM) and Salubrinal (25 µM) to UV-B-exposed HDFs significantly rescue damage response in HDFs as is evident from the decreased expression of p-χH_2_AX in confocal microscopy. Bafilomycin A1 (100 nM) treatment significantly potentiates the expression levels of p-χH_2_AX nuclei in UV-B-exposed HDFs (**Figures 4D, E**). We further carried out the immunofluorescence of DNA damage pathway protein DDB2 in confocal microscopy and found that DDB2 protein expression levels are also significantly upregulated by threefold in UV-B-exposed HDFs compared to control levels. Rapamycin (100 nM) significantly brings the DDB2 level to half whereas Chloroquine (50 µM) treatment drastically increases the expression level of DDB2 protein by 0.2-fold compared to those exposed only to UV-B (**Figures 4F, G**).

### Salubrinal Alleviates DNA Damage and Prevents Immediate ER Calcium Leakage by Improving Cellular Autophagy Levels in UV-B-Exposed HDFs

**Figure 5:**
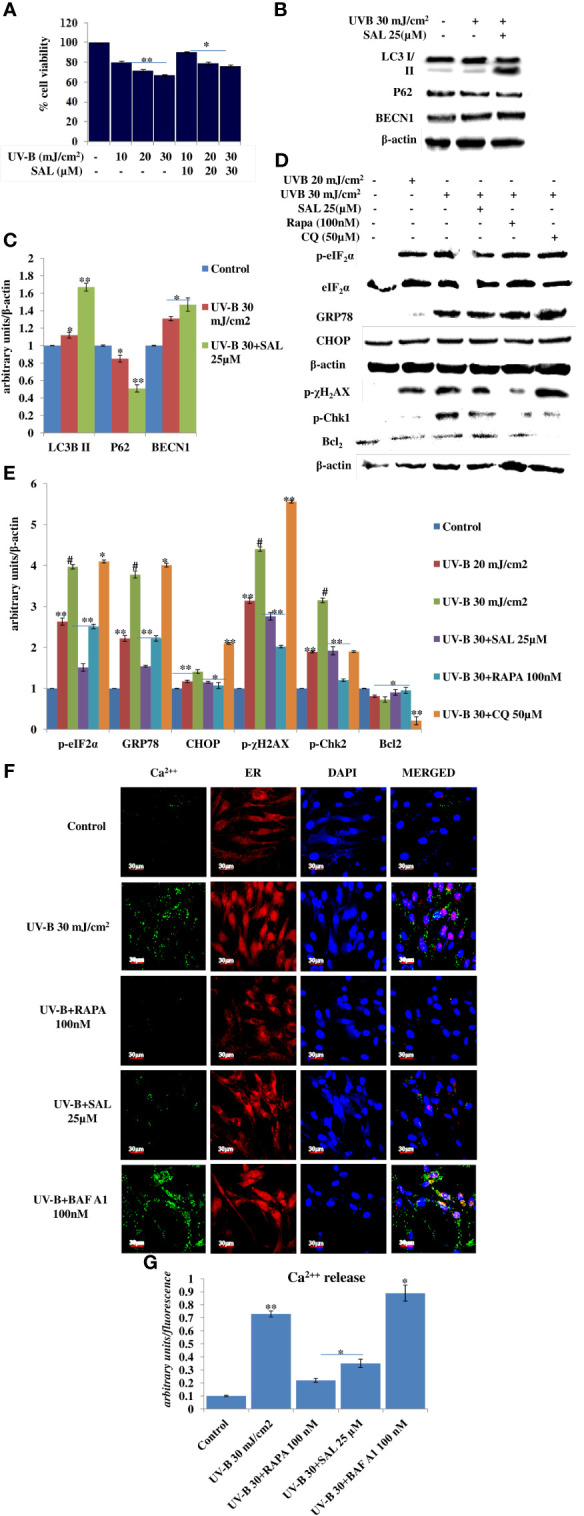
Salubrinal alleviates DNA damage and prevents immediate ER calcium leakage in UV-B-exposed HDFs. **(A)** Cell viability analysis of UV-B 10, 20, and 30 mJ/cm^2^ exposed HDFs in the presence of Salubrinal (10, 20, and 30 µM) in 24 h UV-B post-irradiation. **(B, C)** Western blotting analysis of autophagy marker proteins in Salubrinal (25 µM)-treated HDFs exposed to UV-B. **(D, E)** Western blotting analysis of ER stress and DNA damage response proteins in 20 and 30 mJ/cm^2^ exposed HDFs in the presence of Salubrinal (25 µM), Rapamycin (100 nM), and Chloroquine (50 µM) in 6 h UV-B post-irradiation. **(F, G)** Confocal microscopy analysis of ER calcium depletion in 30 mJ/cm^2^ exposed HDFs in 6 h UV-B post-irradiation in the presence of Rapamycin (100 nM), Salubrinal (25 µM), and Bafilomycin A1 (100 nM) (^*^p ≤ 0.05, ^**^ *p* ≤ 0.01, ^#^ *p* ≤ 0.001 were considered as statistically significant). Western blots were analyzed using Image Lab.exe 3.0.0.39529 and micrographs with ImageJ.exe 1.8.0_172 software.

ER stress response is the immediate manifestation of oxidative stress in UV-B exposure to HDFs. Here, we used Salubrinal, an eIF_2_α phosphatase inhibitor that relieves ER stress response upon UV-B exposure to cells. We first performed the cell viability assay of Salubrinal in UV-B-exposed HDFs and found it cytoprotective in nature. UV-B exposure decreases the cellular viability in HDFs in an intensity-dependent manner in 24 h MTT assay by 20%, 27%, and 35% at 10, 20, and 30 mJ/cm^2^ exposure, respectively (**Figure 5A**). Salubrinal treatment at 10, 20, and 30 µM improves the cellular viability by 1-fold, 0.5-fold, and 0.5-fold in UVB 10 mJ+SAL 10 µM-, 20 mJ+SAL 20 µM-, and UVB 30 mJ+SAL 30 µM-treated HDFs respectively. Furthermore, we checked the expression of autophagy, ER stress, and DDR p-χH_2_AX protein levels in UV-B-exposed HDFs in Western blotting in 24 h upon Salubrinal treatment. UV-B exposure to HDFs induces ER stress response as evident from the expression levels of p-eIF_2_α and upregulates expression of DNA damage sensor protein p-χH_2_AX in Western blotting, but Salubrinal (20 µM) fails to significantly improve the ER stress and damage response events in UV-B-exposed HDFs in 24 h UV-B post-irradiation (**Supplementary Figure S1**). We reduced the UV-B post-exposure time interval to 6 h, because both autophagy and ER stress responses are the initial molecular events following UV-B exposure to HDFs as already reported in our previous study (19). We found that Salubrinal 25 µM improves autophagy response in UV-B-exposed HDFs in 6 h UV-B post-irradiation as is evident from expression levels of LC3B II, P62, and BECN1 in Western blotting (**Figures 5B, C**) as well as augments the punctae dots in GFP-RFP-LC3B puncta assay in confocal microscopy (**Figures 1F, G**). We further found that ER stress is induced upon UV-B exposure to HDFs in an intensity-dependent manner in 6 h UV-B post-irradiation and is significantly alleviated by treatment with Salubrinal 25 µM as evident from the expression of key ER stress response proteins, p-eIF_2_α, GRP78, and CHOP/GAD153. Rapamycin (100 nM) treatment also significantly relieves the UV-B-induced ER stress response whereas Chloroquine (50 µM) fails to rescue the cells from ER stress response in UV-B-exposed HDFs (**Figures 5D, E**). Furthermore, to check whether relieving ER stress response with Salubrinal and improving cellular autophagy response with Rapamycin have any impact on rescuing the UV-B-exposed HDFs from aberrant DDR, we looked for the expression of DNA damage sensor protein p-χH_2_AX and p-Chk1 and found that Salubrinal (25 µM) and Rapamycin (100 nM) significantly alleviate the expression of both p-χH_2_AX and p-Chk1 in Western blotting (**Figures 5D, E**). Salubrinal (25 µM) and Rapamycin (100 nM) treatment also significantly restores the expression levels of anti-apoptotic protein Bcl_2_, whose expression is dwindled upon UV-B 30 mJ/cm^2^ exposure to HDFs, whereas Chloroquine (50 µM) augments the expression of Bcl2 compared to those exposed only to UV-B (**Figures 5D, E**). ER calcium depletion is the immediate molecular event following UV-B exposure to HDFs. Rapamycin (100 nM) and Salubrinal (25 µM) treatment significantly prevents the ER Calcium leakage in UV-B-exposed HDFs; Bafilomycin A1 (100 nM) potentiates the ER calcium depletion from UV-B-exposed HDFs as is evident from confocal microcopy analysis in calcium staining (**Figures 5F, G**).

### Autophagy Blockage *via* P62 Silencing Augments the DDR in UV-B-Exposed HDFs

**Figure 6:**
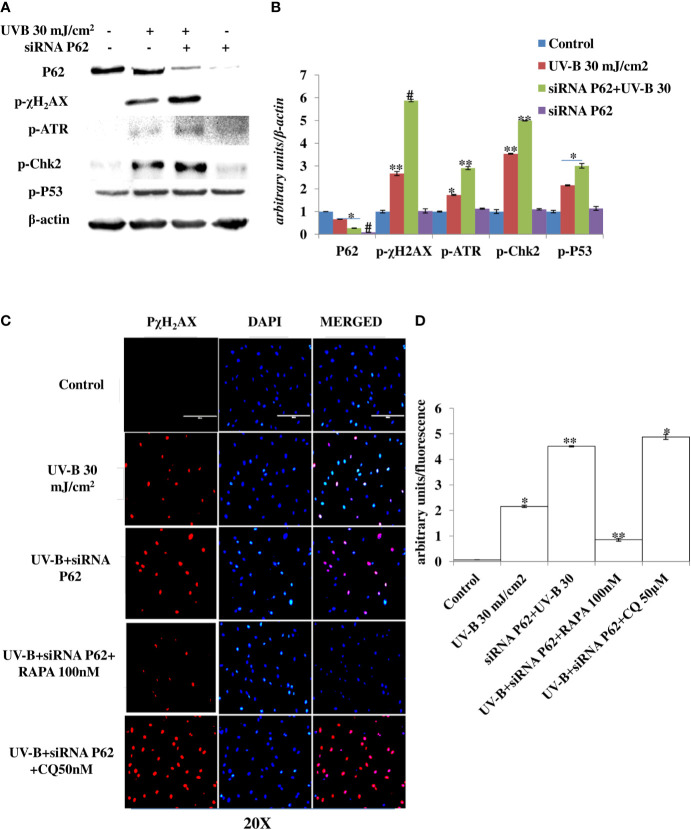
P62 silencing enhances the DNA damage response in UV-B-exposed HDFs. **(A, B)** Western blotting analysis of DNA damage response proteins in siRNA P62-treated HDFs in 6 h UV-B 30 mJ/cm^2^ post-irradiation. **(C, D)** Immunofluorescence analysis of DNA damage sensor protein p-χH_2_AX in siRNA P62-treated HDFs in 6 h UV-B post-irradiation in the presence of Rapamycin (100 nM) and Chloroquine (50 µM) (^*^p ≤ 0.05, ^**^ *p* ≤ 0.01, ^#^ *p* ≤ 0.001 were considered as statistically significant). Western blots were analyzed using Image Lab.exe 3.0.0.39529 and micrographs with ImageJ.exe 1.8.0_172 software.

Autophagy is the main cellular pathway that is activated during stress response in order to restore the normal homeostasis in cells subjected to genotoxic stress. We blocked autophagy response in UV-B-exposed HDFs through silencing autophagy cargo protein P62 to find the impact of autophagy blockage on DDR in 6 h UV-B post-irradiation to HDFs. Western blotting analysis confirmed the silencing of P62 and depicts 95% silencing efficiency. UVB 30 mJ exposure to HDFs shows mild autophagy induction as evident from the downregulation of P62 protein levels by 0.25-fold in Western blotting analysis (**Figures 6A, B**). Furthermore, we found that P62-silenced HDFs reveal enhanced DDR upon UV-B 30 mJ exposure in 6 h post-irradiation as is clear from augmented expression levels of DDR proteins p-χH_2_AX, p-ATR, p-Chk2, and p-P53 in Western blotting analysis (**Figures 6A, B**). P62 siRNA-only-treated cells show negligible effect on the change in expression of DDR proteins in Western blotting analysis compared to control levels. We confirmed our Western blotting results through immunofluorescence in confocal microscopy by looking for the p-χH_2_AX foci and found that UV-B 30 mJ exposure significantly induces the expression of p-χH_2_AX foci in P62-silenced cells compared to those exposed only to UV-B. Rapamycin (100 nM) treatment to P62-silenced HDFs upon UV-B exposure decreases the p-χH_2_AX foci by twofold in immunofluorescence. Chloroquine (50 µM) treatment to P62-silenced cells upon UV-B exposure fails to alleviate the DDR in HDFs as is evident in immunofluorescence (**Figures 6C, D**).

### Autophagy Blockage *via* P62 Silencing Dwindles the Tumor Suppressor PTEN/AKT Pathway in UV-B-Exposed HDFs

**Figure 7:**
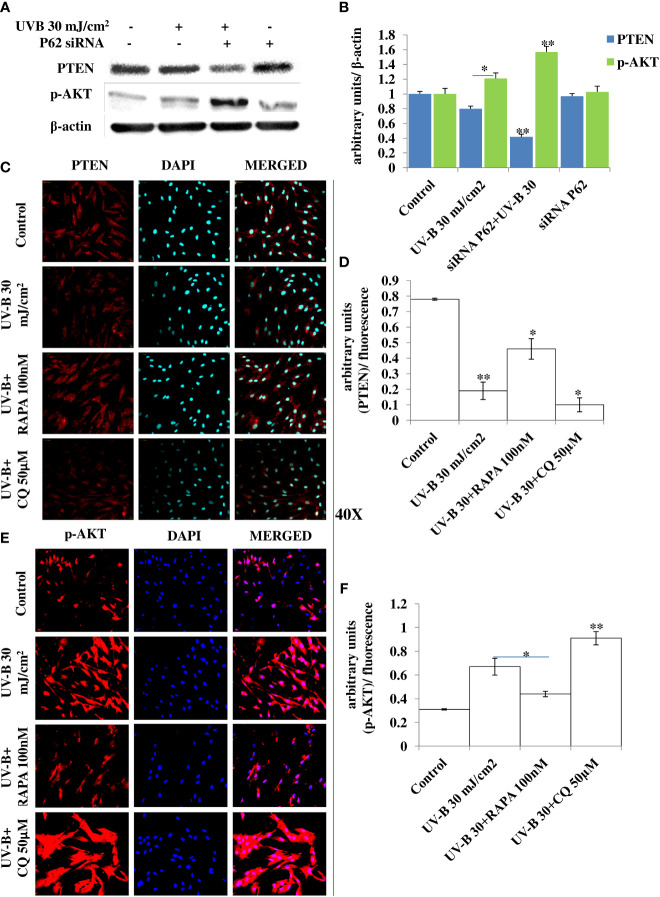
P62 silencing dwindles the PTEN/AKT tumor suppressor signaling axis in UV-B-exposed HDFs. **(A, B)** Western blotting analysis of PTEN/p-AKT protein expression levels in siRNA P62-treated HDFs in 6 h UV-B 30 mJ/cm^2^ post-irradiation. **(C, D)** Immunofluorescence analysis of PTEN protein expression levels in UV-B 30 mJ/cm^2^ exposed HDFs in 6 h UV-B post-irradiation in the presence of Rapamycin (100 nM) and Chloroquine (50 µM). **(E, F)** Immunofluorescence analysis of p-AKT protein expression levels in UV-B 30 mJ/cm^2^ exposed HDFs in 6 h UV-B post-irradiation in the presence of Rapamycin (100 nM) and Chloroquine (50 µM) (^*^p ≤ 0.05, ^**^p ≤ 0.01 were considered as statistically significant). Western blots were analyzed using Image Lab.exe 3.0.0.39529 and micrographs with ImageJ.exe 1.8.0_172 software.

The PTEN/AKT pathway is the main tumor suppressor pathway that promotes cell survival and reduces tumorigenesis in UV-B-induced photo-damage. Here we checked whether blockage of autophagy has any substantial impact on the PTEN/AKT pathway in UV-B 30 mJ exposed HDFs in 6 h UV-B post-irradiation. We found that UV-B irradiation to HDFs significantly downregulates the expression level of PTEN by 0.2-fold and increases the expression of p-AKT protein by 0.25-fold in Western blotting analysis (**Figures 7A, B**). Autophagy blockage *via* P62 silencing significantly downregulates the expression of PTEN by twofold in UV-B+P62-treated HDFs compared to those exposed only to UV-B. We got very interesting results for p-AKT, and the protein expression level of p-AKT is upregulated by 0.4-fold in P62-silenced HDFs compared to those exposed only to UV-B. Similar results were obtained for PTEN and p-AKT in immunofluorescence in confocal microscopy analysis. PTEN protein expression levels are significantly downregulated upon UV-B exposure to HDFs in immunofluorescence. Autophagy activator Rapamycin (100 nM) significantly restores the PTEN expression level by twofold whereas inhibitor of autophagy, Chloroquine (50 µM), could not restore the PTEN expression but adversely augments the expression level of PTEN by onefold compared to those exposed only to UV-B as is evident from immunofluorescence (**Figures 7C, D**). p-AKT expression level in UV-B-exposed HDFs on the other hand is upregulated by twofold in immunofluorescence compared to control levels. Rapamycin (100 nM) treatment significantly brings the expression of p-AKT close to control levels whereas Chloroquine (50 µM) drastically increases the expression of p-AKT in UV-B-exposed HDFs by 0.35-fold compared to those exposed only to UV-B (**Figures 7E, F**).

### Pharmacological Activation of Autophagy Improves the Expression of Cell Cycle Regulatory Proteins in UV-B-Exposed HDFs

**Figure 8:**
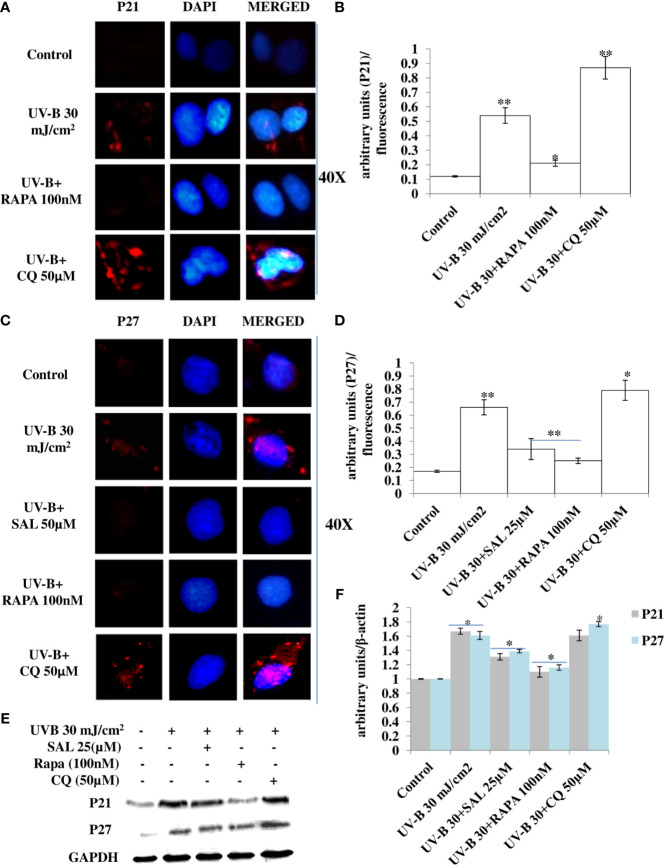
Pharmacological activation of autophagy regulates the expression of cell cycle regulatory proteins in UV-B-exposed HDFs. **(A, B)** Immunofluorescence analysis of P21 protein expression levels in UV-B 30 mJ/cm^2^ exposed HDFs in 24 h UV-B post-irradiation in the presence of Rapamycin (100 nM) and Chloroquine (50 µM). **(C, D)** Immunofluorescence analysis of P27 protein expression levels in UV-B 30 mJ/cm^2^ exposed HDFs in 24 h UV-B post-irradiation in the presence of Salubrinal (25 µM), Rapamycin (100 nM), and Chloroquine (50 µM). **(E, F)** Western blotting analysis of P21 and P27 protein expression levels in UV-B 30 mJ/cm^2^ exposed HDFs in 24 h UV-B post-irradiation in the presence of Salubrinal (25 µM), Rapamycin (100 nM), and Chloroquine (50 µM) (^*^p ≤ 0.05, ^**^p ≤ 0.01 were considered as statistically significant). Western blots were analyzed using Image Lab.exe 3.0.0.39529 and micrographs with ImageJ.exe 1.8.0_172 software.

Cell cycle regulatory proteins play an important role in quality control mechanism and respond to any genotoxic insult and prevent cancer development in cells. In line with this, we checked the effect of pharmacologically stimulated autophagy response on main cell cycle regulator proteins in immunofluorescence through confocal microscopy in 24 h UV-B post-irradiation to HDFs. We found that the expression of P21 protein upregulates in UV-B-exposed HDFs by threefold compared to control levels. Rapamycin (100 nM) treatment significantly improves the expression level of P21 compared to those exposed only to UV-B. Chloroquine (50 µM) on the other hand, drastically increases the expression of P21 in immunofluorescence by 1.5-fold compared to those exposed only to UV-B (**Figures 8A, B**). Similarly, we got augmented expression in the protein levels of P27 in UV-B 30 mJ exposed HDFs by threefold compared to control levels. Salubrinal (25 µM) and Rapamycin (100 nM) treatment brought the expression of P27 to that of control levels, whereas Chloroquine (50 µM) significantly increased the expression of p27 by 0.2-fold compared to UV-B levels in immunofluorescence (**Figures 8C, D**). Similar results were obtained in Western blotting as well wherein UV-B exposure to HDFs increases the expression of P21 and P27 whereas Salubrinal (25 µM) and Rapamycin (100 nM) alleviate the expression of both P21 and P27 whereas Chloroquine (50 µM) augments the UV-B-induced expression of P21 and P27 in Western blotting analysis (**Figures 8E, F**).

### *Atg7* Silencing in an Unexpected Consequence Alleviates the DDR in UV-B-Exposed HDFs

**Figure 9:**
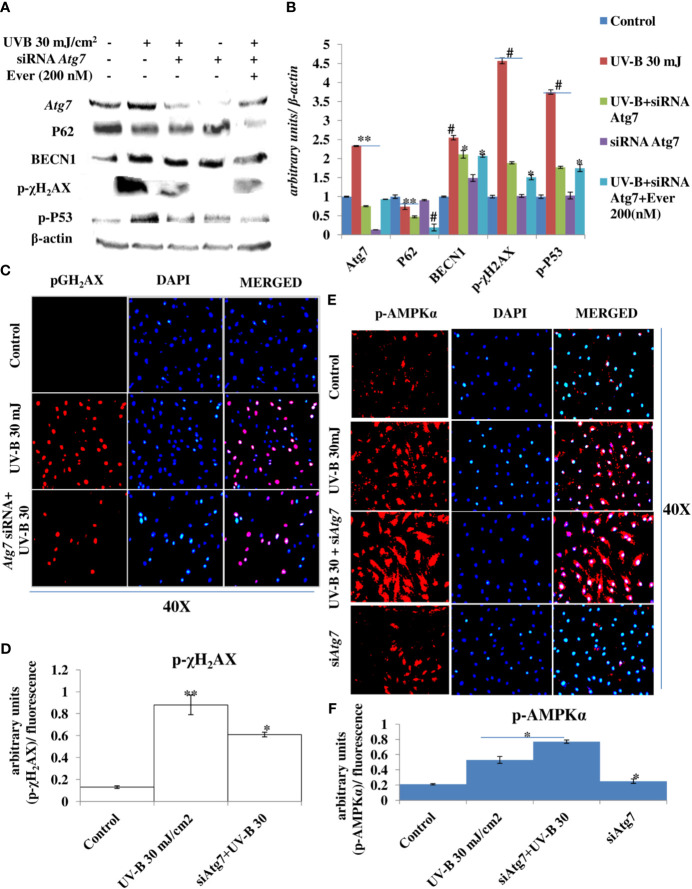
*Atg7* silencing in an unexpected consequence alleviates the DNA damage response in UV-B-exposed HDFs. **(A, B)** Western blotting analysis of autophagy and DNA damage response proteins in siRNA *Atg7*-treated HDFs in 6 h UV-B post-irradiation in the presence of Everolimus (200 nM). **(C, D)** Immunofluorescence analysis of DNA damage sensor protein p-χH_2_AX in siRNA *Atg7-*treated HDFs in 6 h UV-B post-irradiation. **(E, F)** Immunofluorescence analysis of p-AMPKα expression levels in siRNA *Atg7-*treated HDFs in 6 h UV-B post-irradiation (^*^p ≤ 0.05, ^**^ *p* ≤ 0.01, ^#^ *p* ≤ 0.001 were considered as statistically significant). Western blots were analyzed using Image Lab.exe 3.0.0.39529 and micrographs with ImageJ.exe 1.8.0_172 software.

Autophagy-related genes play an important role in the initiation and execution process of autophagy response. Recently, autophagy-related *Atg7* gene deletion has been found to be involved in the suppression of UV-B-induced inflammation and tumorigenesis. To check the specific role of autophagy-related proteins particularly the role of *Atg7* in UV-B-induced photo-damage, we subjected HDFs to *Atg7* silencing in 6 h UV-B post-irradiation. Western blotting analysis confirmed the silencing of *Atg7* with 95% efficiency compared to control levels. UV-B 30 mJ exposure to HDFs increases the protein expression of *Atg7* and BECN1 by 1- and 1.25-fold, respectively, and decreases the protein expression of P62 by 0.5-fold compared to control levels. UV-B exposure to HDFs also induces the expression of key DDR proteins p-χH_2_AX and p-P53 by 5- and 4.5-fold, respectively. *Atg7* silencing in UV-B-exposed HDFs significantly alleviates the DDR as is evident from decrease in the protein expression levels of DDR proteins p-χH_2_AX and p-P53 in Western blotting by 2.5- and 2-fold, respectively. *Atg7*-only silenced HDFs show negligible effect on the modulation in expression level of autophagy and DNA damage marker proteins in Western blotting analysis. Everolimus (200 nM) treatment to *Atg7*-silenced HDFs upon UV-B exposure also decreases the expression levels of DDR proteins compared to those exposed only to UV-B but not compared to *Atg7*+UV-B-exposed cells (**Figures 9A, B**). We confirmed our Western blotting results through immunofluorescence by looking out for p-χH_2_AX foci and found that UV-B exposure to HDFs significantly induces the p-χH_2_AX damage foci by 3-fold compared to control levels but are significantly alleviated in *Atg7*-silenced UV-B-exposed HDFs by 0.35-fold compared to that of those exposed only to UV-B (**Figures 9C, D**). Furthermore, confocal microscopy analysis of p-AMPKα protein expression levels in 6 h UV-B post–irradiation to HDFs show that autophagy protein *Atg7* silencing significantly enhances the p-AMPKα expression levels in UV-B-irradiated HDFs compared to those exposed only to UV-B (**Figures 9E, F**).

## Discussion

Skin aging is a dynamic process and depends on both intrinsic factors such as genetics and hormones, as well as extrinsic factors including UV radiation and environmental pollutants (28). UV radiation in particular is considered the most crucial factor for skin aging due to the process known as photoaging (29). Incidences of skin cancer have increased in recent years, possibly due to increased exposure to solar UV radiation because of the depletion of ozone layer in the stratosphere (30). UV-B irradiation to skin stimulates diverse cellular and molecular responses like inflammation, reactive oxygen species (ROS) formation, and endoplasmic reticulum (ER) stress response that ultimately leads to autophagy induction in skin cells aimed at restoring the cellular homeostasis upon encountering insult. Acute and chronic exposure of UV-B to skin lead to various perturbations that leads to aging-related signal transduction amplification, ultimately resulting in skin damage and photoaging (31). UV-B radiation is considered as the most mutagenic component of UV spectrum reaching the earth’s surface and causes DNA damage in the form of cyclobutane pyrimidine dimers (CPD) and pyrimidine-(6-4)-pyrimidone photoproducts (6,4PP) affecting DNA integrity and tissue homeostasis, and causes mutations in oncogenes and in tumor suppressor genes (32). Cells have, in defense, a natural, inbuilt, and well-established molecular response system known as DDR that checks any mutagenic insult to genome and repairs it immediately through the Nucleotide Excision Repair system, to prevent tumor development and cancer progression in cells. Any unrepaired part can lead to abnormal cell growth, increasing the risk of cancer (33). Autophagy is a cellular catabolic process that has roles in sensing nutrient stress during starvation conditions and cleanses cellular debris generated as a metabolic by-product (34). Dysfunction of the autophagic process is known to have a role in the development of human chronic pathologies, such as metabolic, cardiovascular, and neurodegenerative diseases, and in cancer as well. Comprehensive research is ongoing to discover new therapeutic strategies and agents able to modulate the autophagic process. Special attention is given in particular to understand the complex role of autophagy in disease pathogenesis. Research efforts are now focused on understanding the context-dependent roles of autophagy and on the evaluation of the pharmacological effects of autophagy signaling in a more in-depth and mechanistic way (35). Furthermore, the development of skin aging is associated with several molecular changes including the accumulation of DNA damage, genome instability, epigenetic dysregulation, mitochondrial dysfunction, inflammation, extracellular matrix degradation, loss of proteostasis, ER stress, and autophagy dysfunction (36). Many of these genome alterations are directly associated with cellular damage and senescence, which is one of the hallmarks of skin aging. Recent evidence from autophagy research in UV-B-induced skin DNA damage has provided novel insights and has been found to play a positive and pivotal role in DNA damage recognition by nucleotide excision repair and also controls p38 activation to promote cell survival under genotoxic stress conditions (5, 37). In another similar study, it has been found that autophagic UVRAG promotes UV-induced photolesion repair by activation of the CRL4 (DDB2) E3 Ligase (38) citing the positive role of autophagy in regulating UV-B-induced damage response in skin. We have recently reported that a natural product-based anti-oxidant molecule, Glycyrrhizic acid, alleviates oxidatively induced DNA damage through improving cellular autophagy levels in primary human dermal fibroblasts (19). Furthermore, recent works have revealed that genotoxic stress is a trigger for autophagy and autophagy regulates repair of UV-induced DNA damage. Previously, it has been revealed that knockdown of autophagy genes such as AMPK, *Atg5, Atg7, Atg12*, and *Atg14* impairs the repair of UVB-induced DNA damage (39). Despite these preliminary studies conducted so far in demystifying the role of autophagy in UV-B-induced DDR, the precise role of autophagy in regulating UV-B-induced genotoxic stress is yet to be ascertained and warrants further studies to unravel the facts. In line with these findings, we planned the current study and hypothesized that autophagy might be playing a very crucial role in regulating UV-B-induced DDR. Our results suggest that UV-B irradiation induces impaired autophagy response in a dose-independent manner as is evident from the Western blotting analysis of key autophagy marker proteins not corresponding to induction of healthy autophagy response in cells (**Figures 1A–E**). Induction of impaired autophagic flux response upon UV-B 30 mJ/cm^2^ exposure to HDFs was confirmed through GFP-RFP-LC3B puncta assay in confocal microscopy where we obtained appreciable red puncta dots, indicative of autolysosomes compared to yellow puncta dots indicative of autophagosomes in Rapamycin- and Salubrinal-treated cells showing enhanced autophagy flux upon UV-B exposure to HDFs compared to those exposed only to UV-B (**Figures 1F, G**). ELISA-based results reveal that DNA photo-adducts (CPD and 6,4PP) are the immediate by-products of oxidative damage in UV-B-exposed HDFs in 6 h UV-B post-irradiation. Improving autophagic flux with Rapamycin (100 nM) and relieving UV-B-induced ER stress response with Salubrinal (25 µM) significantly alleviate the induction of both CPD and 6,4PP in UV-B-exposed HDFs. Autophagy inhibitor [Chloroquine (25 µM)] treatment of HDFs on the other hand augments the induction of both CPD and 6,4PP in UV-B-treated HDFs (**Figure 2A**), indicating that autophagy induction positively regulates the formation of DNA photo-adducts in UV-B-induced photo-damage. We confirmed these findings by silencing the autophagy cargo protein P62 and found that it significantly increases the induction of photo-adducts, indicating that autophagy plays a very critical role in regulating the UV-B-induced photo-damage response (**Figure 2B**). Similar effects were obtained in immunofluorescence on checking the effect of improving cellular autophagy levels on CPD induction, further revealing that pharmacological activation of autophagy positively regulates the DDR in UV-B exposure to HDFs (**Figures 2D, E**). Our results further reveal that Rapamycin (100 nM) treatment of HDFs has no significant effect on improving the cellular viability in acute dose (UV-B 30 mJ/cm^2^) exposed HDFs whereas Chloroquine 50 µM treatment significantly reduces the cell viability from 65% in UV-B 30 mJ/cm^2^ only exposed to 60% in UV-B 30+CQ (50 µM) treated, indicating that inhibition of autophagy potentiates the cell death effect in UV-B-exposed HDFs (**Figure 2C**). It is a fact that UV-induced skin damage triggers cascade of response signaling pathways, including cell cycle arrest, DNA repair, and, if left unrepaired, can lead to apoptotic events (40). Oxidatively induced DDR is the hallmark of UV-B-induced skin photo damage that ultimately leads to genotoxic stress response in skin (41). We found that UV-B treatment of HDFs induces TUNEL-positive cells in an intensity-dependent manner in 6 h UV-B post-irradiation. Rapamycin treatment (100 nM) significantly alleviates TUNEL-positive cells in UV-B 30 mJ/cm^2^ exposed HDFs whereas Chloroquine treatment (50 µM) of HDFs increases TUNEL-positive cells compared to those exposed only to UV-B (**Figures 3A, B**). AO/EtBr co-staining also reveals increased ratio in early to late apoptotic nuclei in UV-B-exposed HDFs indicating nuclear damage upon UV-B exposure. Salubrinal (25 µM) and Rapamycin (100 nM) treatment significantly alleviates the fluorescence of apoptotic nuclei to that of control levels whereas Chloroquine (50 µM) treatment increases the apoptotic nuclei compared to UV-B levels, indicating that Salubrinal and Rapamycin have a positive role in regulating the UV-B-induced apoptosis whereas Chloroquine potentiates the damage by increasing the late apoptotic cells, but not necrotic cells. These results clearly indicate that oxidative stress, ER stress, and autophagy are intricately interconnected and autophagy has a very critical role in alleviating stress response in UV-B-exposed HDFs (**Figures 3C, D**). Moreover, oxidative stress induced upon UV-B exposure to skin cells also induces autophagy and ROS is the main oxidative damage-causing agent in UV-B-exposed cells (42). We found that treatment with Salubrinal and Everolimus significantly alleviates the ROS levels produced in response to UV-B exposure to skin cells, indicating that improving autophagy has a role to play in regulating oxidative stress response and that ER stress and oxidative stress are mutually related to each other, disturbing the cellular homeostasis (**Figures 3E, F**). Intense UV-B exposure to HDFs induces genomic damage and cell cycle arrest, which is critical at providing ample time gap for DNA damage recognition and subsequent execution of repair process. UV-induced DNA damage activates the sensors ataxia telangiectasia mutated (ATM) and ataxia telangiectasia and Rad3-related (ATR) to trigger cell cycle arrest *via* p53 stabilization (43) and also phosphorylates checkpoint kinase 1 (Chk1) to activate checkpoints at the G1, S, and G2/M phases. Damage-related protein DDB2 has been shown to facilitate the recruitment of ATM and ATR to sites of DNA damage and promote the activation of cell cycle arrest pathways (44–46). We found that UV-B exposure to skin cells induces DDR and is both a time- and intensity-dependent event as is evident from the Western blotting analysis of DDR proteins (**Figures 4A, B**). The expression levels of p-AKT, which has a crucial role in pro-survival signaling and also inhibits apoptosis in UV-B response, are significantly downregulated initially at low UV-B intensity but increase in 24 h UV-B post-irradiation exposure to HDFs (**Figures 4A, C**). p-χH_2_AX foci are immediately induced upon DNA damage in cells, sensing damage and facilitating repair process. Here, we found that UV-B 30 mJ/cm^2^ exposure to HDFs induces the expression of p-χH_2_AX foci in immunofluorescence. Rapamycin (100 nM) and Salubrinal (25 µM) treatment rescues the DNA damage and accelerates the repair process in UV-B-exposed HDFs as is evident from the decreased expression of p-χH_2_AX, whereas Bafilomycin A1 (100 nM) treatment shows enhanced expression levels of p-χH_2_AX nuclei in UV-B 30 mJ/cm^2^ exposed HDFs, indicating that autophagy positively regulates the damage response and repair process in UV-B exposure to skin (**Figures 4D, E**). Immunofluorescence of DDB2 also reveals upregulated levels of expression in UV-B 30 mJ exposed HDFs. Here, Rapamycin (100 nM) also significantly brings the DDB2 level to that of control levels whereas Chloroquine (50 µM) treatment drastically increases the expression level of DDB2 protein compared to UV-B-exposed HDFs (**Figures 4F, G**), indicating that DDR dwindles and subsequent repair processes are accelerated if autophagy levels are improved in UV-B-exposed HDFs and the pro-survival capacity of cells is enhanced due to clearing of damage incurred due to UV-B exposure to HDFs.

Previously, we have reported that oxidative stress-mediated Ca^2+^ release manifests ER stress leading to unfolded protein response in UV-B-irradiated human skin cells (18). In another study, it was reported that Salubrinal, an eIF_2_α phosphatase inhibitor, protects human skin fibroblasts against UVB-induced cell death by blocking ER stress and regulating calcium homeostasis (47). Many other studies reported the diverse roles of Salubrinal on autophagy in different pathological conditions (48, 49) but not in skin photo-damage response. Here, we found that Salubrinal (10–30 µM) significantly improves the cellular viability in MTT assay in UV-B 10–30 mJ/cm^2^ exposed HDFs (**Figure 5A**). Salubrinal (25 µM) also improves cellular autophagy levels in 6 h UV-B post-irradiation to HDFs (**Figures 5B, C**, **Figures 1F, G**). Furthermore, Salubrinal significantly alleviates ER stress response as well as DNA damage and accelerates repair process in 6 h UV-B post exposure to HDFs (**Figures 5D, E**) but not in 24 h UV-B post-irradiation (**Supplementary Figure S1**). ER calcium leakage is the immediate post-event following UV-B exposure to HDFs as reported earlier (18). We found that UV-B-mediated ER calcium leakage is significantly prevented by Rapamycin (100 nM) and Salubrinal (25 µM) treatment to UV-B-exposed HDFs in 6 h UV-B post-irradiation, whereas Bafilomycin A1 (100 nM) fails to prevent the ER calcium depletion in calcium staining in microscopic analysis (**Figures 5F, G**).

Previous studies have reported that P62 modulates the intrinsic signaling in UVB-induced apoptosis (50) and that autophagy performs a crucial role in promoting cell survival under genototoxic stress and helps in preventing tumorigenesis. Degradation of P62 has been found to mediate an important tumor-suppressive function of autophagy, and autophagy-deficient conditions have shown enhanced P62 accumulation and act as a signaling hub by forming interactions with a number of pro-tumorigenic proteins, thereby promoting tumorigenesis (51). Here, we found that autophagy blockage *via* P62 silencing shows enhanced DNA damage and defective repair in 6 h UV-B 30 mJ post-irradiation as is clear in Western blotting analysis of DDR proteins (**Figures 6A, B**) and similar results were obtained in immunofluorescence of p-χH_2_AX foci in confocal microscopy, which significantly increase in P62-silenced cells compared to those exposed only to UV-B. Rapamycin (100 nM) decreases the expression of damage sensor p-χH_2_AX to that of control levels whereas Chloroquine (50 µM) significantly increases the expression of p-χH_2_AX in immunofluorescence than in UV-B alone and P62-silenced HDFs irradiated with UV-B, indicating that autophagy induction regulates the UV-B-induced damage and also impacts upon repair process and imparts pro-survival capability to UV-B-exposed HDFs, thereby reducing chances of cancer development (**Figures 6C, D**). Earlier, it has been reported that PTEN, which otherwise activates autophagy, is inhibited by Sestrin 287 and, in response to UV-B exposure, impairs the GG-NER by downregulating XPC transcription. However, it is still elusive to conclude whether downregulation of autophagy has a significant role in PTEN-regulated DNA damage repair in response to UV-B exposure to skin cells (52, 53). The PTEN/AKT pathway is the crucial tumor suppressor pathway that promotes cell survival under genotoxic stress, and earlier studies have found that ERK/AKT-dependent PTEN suppression promotes survival of epidermal keratinocytes under UV-B exposure. Our results are in complete agreement with these initial findings as P62 silencing disturbs the PTEN/p-AKT pathway in UV-B-exposed HDFs in 6 h UV-B post-irradiation by decreasing the PTEN protein expression level but increases the p-AKT levels compared to UV-B (**Figures 7A, B**). Similar results were obtained in immunofluorescence of PTEN and p-AKT (**Figures 7C–F**, respectively), which indicate that autophagy also regulates tumor suppressor pathway under UV-B-induced genotoxic stress. Furthermore, Rapamycin (100 nM) improves the PTEN/p-AKT-mediated tumor suppressor pathway whereas Chloroquine (50 µM) potentiates the UV-B response to the PTEN/p-AKT pathway in UV-B-exposed HDFs in microscopy backing our silencing results. Furthermore, the massive accumulation of DNA lesions within the cells under different genotoxic stimuli interferes with the replication process, prompting cells to stop division and to repair damaged DNA (54). Cell cycle regulator proteins play a very important part in the quality control of cells and in sensing any external insult and preventing mutations. We found that Salubrinal (25 µM) and Rapamycin (100 nM) improve the fate of cell cycle regulator proteins P21 (**Figures 8A, B**) and P27 (**Figures 8C, D**) in immunofluorescence as well as in Western blotting (**Figures 8E, F**) in 24 h UV-B post-irradiation by regulating autophagy response whereas Chloroquine (50 µM) further worsens the damage regulation by increasing the expression of P21 and P27, which indicates that autophagy also plays its part in regulating cell cycle under genotoxic stress conditions.

Earlier studies have revealed an unexpected consequence of *Atg7* gene deletion in the suppression of UVB-induced inflammation, and tumorigenesis and epidermis-specific deletion of *Atg7* have been found to protect against UVB-induced sunburn, vascular permeability, and skin tumorigenesis. Moreover, *Atg7* deletion has been found to regulate UVB-induced skin tumorigenesis by regulating the AMPK and ER pathways (55). Our results are in complete agreement with these previous findings because *Atg7* silencing in UV-B-exposed HDFs alleviates the DDR and accelerates the repair process induced in 6 h UV-B post-irradiation to HDFs as is evident from the Western blotting analysis of key DNA damage marker proteins. Everolimus (200 nM) also rescues the HDFs from UV-B-induced DNA damage under *Atg7* silencing conditions but not as in *UV-B+Atg7*-silenced cells exposed to UV-B (**Figures 9A, B**). Similar results were obtained in immunofluorescence by looking for p-χH_2_AX expression levels that are significantly alleviated in *Atg7*-silenced HDFs compared to those exposed only to UV-B (**Figures 9C, D**) demonstrating an unexpected consequence of *Atg7* silencing and its role in the suppression of UVB-induced DNA damage and in augmenting the repair process. Furthermore, the expression levels of p-AMPKα in *Atg7*-silenced HDFs irradiated with UV-B also increase significantly (**Figures 9E, F**), which is in complete agreement to what the role of *Atg7* has been stated previously, and this activation of p-AMPKα is likely an adaptive response to multiple stresses caused by autophagy deficiency mediated by *Atg7* silencing leading to ER accumulation and subsequent reduction in ER stress, but warrants further studies to clearly understand the role of the autophagy process, particularly the contrasting role of *Atg7* in the regulation of UV-B-induced genotoxic stress response in skin.

## Conclusion

The above findings provide critical insights that indicate the regulatory and functional role of pharmacological activation of autophagy in the regulation of UV-B-induced skin photo-damage and subsequent repair process. These findings further reveal that cellular autophagy levels are critical in sensing and repairing UV-B-induced DNA damage and are crucially involved in DDR under UV-B-induced photo-damage conditions. Oxidative stress–ER stress and DDR mechanisms are tightly regulated, and autophagy is involved in the regulation of all the three pathways and promotes pro-survival capacity of cells under genotoxic stress conditions notably under UV-B-induced DNA damage. Moreover, our findings support the potential role of the autophagy pathway to be explored as a promising therapeutic strategy against UV-B-mediated photo-damage disorders but warrant further studies to clearly demystify the molecular association existing between autophagy and DDR and repair process under genotoxic stress conditions.

## Data Availability Statement

The original contributions presented in the study are included in the article/**Supplementary Material**. Further inquiries can be directed to the corresponding author.

## Author Contributions

SU performed the major experiments. NS and LN performed the high-throughput experiments. MT and GD performed the TUNEL assay experiment. ST conceived and developed the hypothesis, supervised the research work, and arranged the research funding for the work. SU and ST planned the experiments and analyzed the data. SU and ST wrote the manuscript. SA and SR helped in manuscript editing. All authors contributed to the article and approved the submitted version.

## Funding

Authors acknowledge the financial assistance by Director CSIR-Indian Institute of Integrative Medicine, Jammu vide project No. MLP-1003 and by Department of Biotechnology (DBT), Ministry of Science and Technology, New Delhi, India vide project No. GAP-2166. Senior Research Fellowship (SRF) to author SA by the Department of Science and Technology (DST), Ministry of Science and Technology, New Delhi, India Vide Letter No. IF-160982 is acknowledged. Senior Research Fellowship (SRF) to authors LN, MT, and GD by University Grants Commission (UGC) New Delhi, India and to NS by CSIR, New Delhi, India is acknowledged. Junior Research Fellowship to authors SR by Department of Science and Technology (DST), Ministry of Science and Technology, New Delhi, India and to SA by University Grants Commission (UGC) New Delhi is acknowledged.

## Conflict of Interest

The authors declare that the research was conducted in the absence of any commercial or financial relationships that could be construed as a potential conflict of interest.

## Publisher’s Note

All claims expressed in this article are solely those of the authors and do not necessarily represent those of their affiliated organizations, or those of the publisher, the editors and the reviewers. Any product that may be evaluated in this article, or claim that may be made by its manufacturer, is not guaranteed or endorsed by the publisher.
