# Supplementary material for: Pharmacological Activation of Autophagy Restores Cellular Homeostasis in Ultraviolet-(B)-Induced Skin Photodamage
Source: Front Oncol. 2021 Aug 2;11:726066. doi: 10.3389/fonc.2021.726066 (PMC8366585; doi:10.3389/fonc.2021.726066)

Supplementary Figure 1

Western blotting analysis of Salubrinal (20µM) treated HDFs exposed to different doses of UV-B (10, 20 & 30mJ/cm²) showing no rescue of ER stress and DNA damage response in 24h UV-B post-irradiation to HDFs.

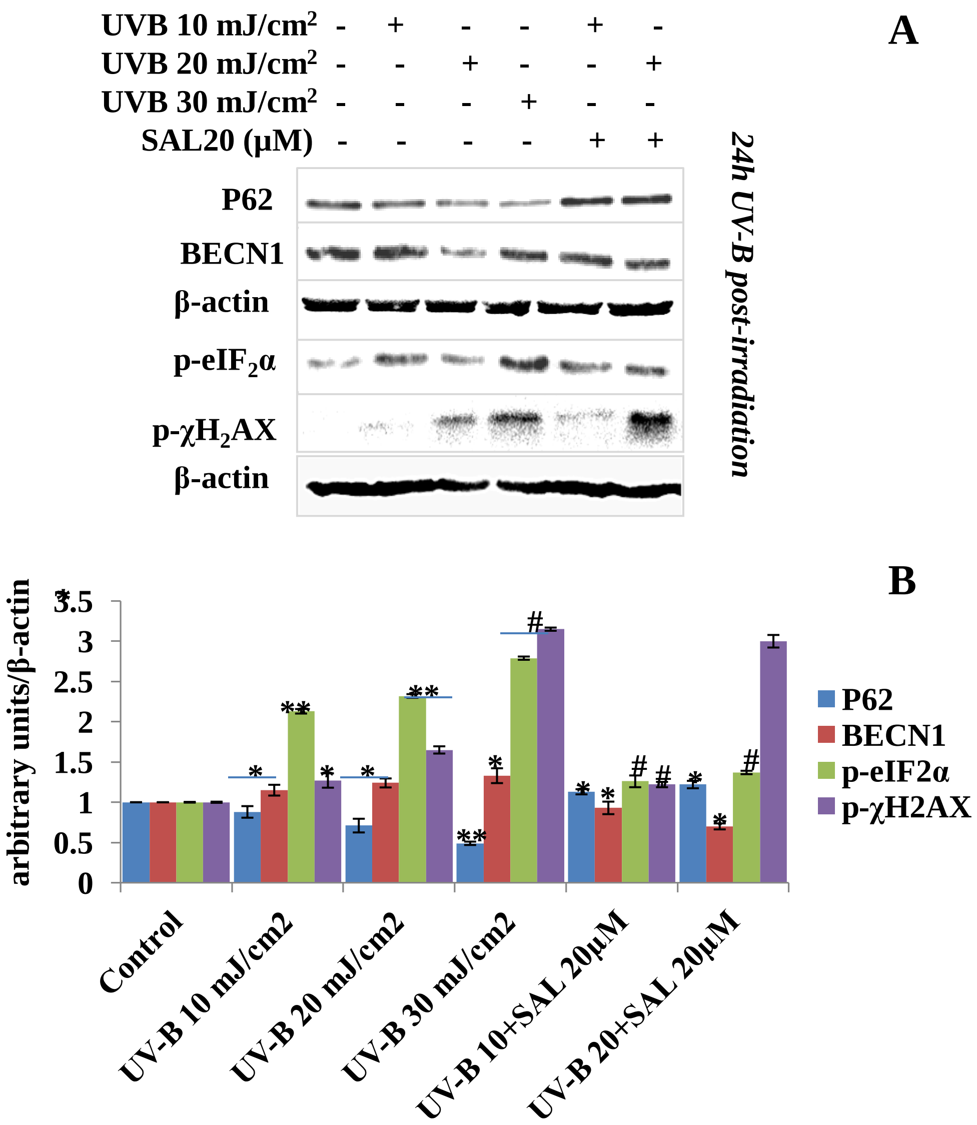

Supplement: Supplementary file 1 [file DataSheet_1.pdf]
